# Comparison of mothers’ perceptions of hunger cues in 3-month-old infant under different feeding methods

**DOI:** 10.1186/s12889-023-15325-3

**Published:** 2023-03-07

**Authors:** Fenghua Zhao, Yijie Sun, Yue Zhang, Tao Xu, Nianrong Wang, Shuangqin Yan, Ting Zeng, Fenghua Zhang, Jie Gao, Qing Yue, Scott Rozelle

**Affiliations:** 1 National Center for Women and Children’s Health, Chinese Center for Disease Control and Prevention, Beijing, 100081 China; 2 Chongqing Health Center for Women and Children, Chongqing, 401147 China; 3 Ma’anshan Maternal and Child Health Hospital, Anhui, 243011 China; 4 Liuzhou Maternity and Child Healthcare Hospital, Guangxi, 545001 China; 5 Qingdao Maternal and Child Health and Family Planning Service Center, Shandong, 266072 China; 6 Stanford University Freeman Spogli Institute for International Studies, Stanford, CA 94305-6055 USA

**Keywords:** Infants, Hunger cues, Breastfeeding, Responsive feeding, Mothers

## Abstract

**Background:**

Mothers’ perception of infant hunger cues is a critical content of responsive feeding, which is central to the promotion of early childhood development. However, only a few studies have examined responsive feeding in China, especially lacking the studies on perceptions of infant hunger cues. Consider the cultural differences, the aim of this study was to describe the perceptions of infant hunger cues of Chinese mothers for infants aged 3 months, and explore the relationship between maternal perceptions of infant hunger cues and different feeding methods.

**Methods:**

A cross-sectional study was conducted with a sample of 326 mothers of healthy 3-month-old infants, including 188 exclusive breastfeeding (EBF) mothers and 138 formula feeding (FF) mothers. It was implemented in four provincial and municipal maternal and child health hospitals. The mothers’ perceptions of infant hunger cues were surveyed by self-reporting questionnaires. Chi-square tests and logistic analysis were applied to analyze the differences in maternal perceptions of infant hunger cues, including the number of hunger cues and the specific cues, between EBF group and FF group by controlling sociodemographic variables and the daily nursing indicators.

**Results:**

We found that a higher proportion of EBF mothers could perceive multiple hunger cues (≥ 2) than FF mothers (66.5% vs.55.1%). For specific cues, the EBF mothers had higher perceptions of infant’s “hand sucking” (67.6% vs. 53.6%) and “moving head frantically from side to side” (34.6% vs. 23.9%), all *p* < 0.05. Regression analysis revealed that EBF might support mothers to perceive infant hunger cues than FF mothers, with the number of infant hunger cues (OR = 1.70, 95% CI: 1.01–2.85), “hand sucking” (OR = 1.72, 95% CI: 1.04–2.87), “moving head frantically from side to side” (OR = 2.07, 95% CI: 1.19–3.62). The number of infant hunger cues perceived by mothers was also associated with their educational level and family structure.

**Conclusion:**

EBF mothers of 3-month-old infants may be more likely to perceive infant hunger cues than FF mothers in China. It is necessary to increase the health education about infant hunger and satiety cues to caregivers in China, especially among mothers with lower education levels, mothers living in nuclear families, and FF mothers.

## Introduction

In recent years, responsive feeding has attracted considerable attention due to its significance in promoting infants health [1– 6]. Responsive feeding is defined as “the interaction between the child and the caregiver” in a feeding context [1], and is related to on-demand feeding and baby-led feeding [7]. Its definition is similar to Mary Ainsworth’s definition of parental sensitivity [8, 9] as a parent’s ability to (1) notice child signals, (2) interpret these signals correctly, and (3) respond to these signals promptly and appropriately. Both responsive feeding and parental sensitivity involve the accurate perception of infant cues.

Empirical research has confirmed the importance of maternal perceptions of infant hunger cues for promoting healthy child outcomes. Several studies have found that mothers who promptly perceive and respond appropriately to their infant hunger cues reduce the risk of overfeeding, rapid weight gain or malnutrition, and even stunting [4, 10–13]. Maternal perceptions of hunger cues in infancy also may influence whether the infant becomes over- or underweight during infancy and toddlerhood [14]. In addition, raising awareness of these cues with mothers may encourage more responsive and positive mealtime interactions [15, 16]. This facilitates emotional linkage and good attachment relationships between parents and infants, thereby promoting cognitive ability and psycho-behavioral development in infants [3]. Therefore, it is necessary to understand the parents’ perception of infant hunger and satiety cues to lay the foundation for achieving responsive feeding.

Mothers’ perception of infant hunger cues is an essential aspect of achieving responsive feeding [7, 15]. Previous studies have shown that infants can self-regulate their energy intake based on their needs [17–20] and that this ability to self-regulate is best promoted by feeding practices in which the caregiver responds to infant hunger or satiety cues [1, 4, 21]. Infant hunger cues include putting their hand in their mouth, increasing physical activity, mouth opening or closing, moving their head frantically from side to side, crying and so on [10, 22–25]. Infants signal hunger through their body movements, facial expressions, and vocalizations [26]. These hunger cues are typically subtle (e.g., putting hand in mouth) in the early stages of hunger and gradually escalate until the cues are perceived and responded to by the mother [25, 27]. Research suggests parental responsiveness to children’s hunger and satiety cues is critical for developing healthy eating habits [4, 14]. However, most studies focus on infants after 6 months [15, 28–30], and there are few literature studies on infants aged 3 months [8]. Exploring mothers’ perception of early infant hunger cues may provide insight into responsive feeding practices during lactation.

The literature has identified various factors that may influence infant hunger cues and parental perception of these hunger cues, including social demography, feeding methods and parenting behavior. Some studies have pointed out maternal perceptions associated with income [31], maternal education level, mother’s country of origin, maternal BMI [23], infant age, infant gender [32] and minority ethnicity [33, 34], history of breastfeeding [23], breast milk intake, sleep patterns and feeding methods [23, 35, 36]. Parenting experience is associated with maternal age and the number of children [37, 38]. The family structure [39] and daily nursing may also influence parenting practices. Therefore, these factors may be related to the mothers’ perception of these hunger cues. Shloim et al. (2015) has shown that breastfeeding mothers of the 2 to 6-month-old infants, compared to FF mothers, can better understand infant hunger cues and provide a more positive feeding experience [36]. Cultural contexts have been shown to have important effects on family care [40], which may also be an effect on mothers’ perception of infant hunger cues [41]. Unfortunately, there is still a lack of research in the current Chinese literature on maternal perception of infant hunger cues. Few studies has examined mothers’ perception of infant hunger cues in China. It is necessary to investigate the Chinese mothers’ perception of infant hunger cues, which can encourage mothers to better understand how to perceive infant hunger cues and respond promptly, and may have reference value for other Southeast Asian countries.

This study is part of the study of mother-infant interaction under different feeding methods [42, 43], which videotaped the feeding progress of mother-infant dyads. However, we focused on analyzing perceptions of infant hunger cues by mothers of 3-month-old infants in this study. The main goals were as follows: (1) to understand Chinese maternal perceptions of hunger cues of 3-month-old infants. (2) to evaluate the association between feeding methods and maternal perceptions of infant hunger cues. Therefore, we hypothesized that feeding methods and other factors may be related to maternal perceptions of infant hunger cues. This is helpful for child health providers to guide the development of responsive feeding for parents.

## Methods

### Design and sampling

A cross-sectional survey approach was used. The survey recruited participants at Maternal and Child Health Hospitals (MCHs: abbreviation of Maternal and Child Health Care Institutions) in 4 cities located in 4 provinces in China from January to December 2019, including Ma’anshan in central China, Liuzhou in southern China, Chongqing in western China, and Qingdao in eastern China. These cities are geographically widespread. The sample size for this study was computed as 270, according to the calculation based on the 66% of mothers could perceive hand sucking as hunger cue [23] (lack of reports of Chinese population), α = 0.05, permissible error = 0.08, and stratified by feeding methods. Each group(EBF group and FF group)was determined to contain at least 135 mother-child dyads.

To recruit study participants, the research team connected with the MCHs who participated in the study. The child healthcare doctors of these hospitals, trained by the project team, approached and interviewed mothers whose healthy infants were undergoing routine health examinations at the hospital at the time of the survey to determine if they met the inclusion criteria and whether they would like to participate. The inclusion criteria were as follows: (a) the family had a local household registration or had resided in the city for more than six months; (b) the infant was full term (≥ 37 weeks); (c) the infant’s birth weight was ≥ 2500 g; (d) the infant had been fed only fed by breastmilk for the last 24 h and did not supplement with any food except drugs and vitamins (for infants in the EBF group),or had not been breastfed for the last 24 h (for infants in the FF group). All infants were required to a single child with either EBF or FF was required to account for more than 90% of total intake for the first three months after birth. The above information was obtained through interview, and mothers were given ample time to recall and consider their responses. The following were excluded: (a) infants with health issues that might affect feeding abilities (e.g. swallowing); (b) mothers or infants with serious diseases, including chronic health problems that would affect the growth and/or development of the infant or complications during pregnancy and/or childbirth of the mother; (c) mothers who did not have normal reading and writing skills.

### Measurements

Variables assessed in this study were obtained from self-report questionnaires designed by the study team, including general sociodemographic data, nursing-related variables, and variables of maternal perceptions of infant hunger cues.

Sociodemographic variables included infant gender, ethnicity, maternal age, maternal education level, family structure, and one-child status. Nursing-related variables included “whether the mother is the primary caregiver”, “whether the baby sleeps with mother”, feeding interval, and feeding duration.

#### Measures the mother’s perceptions of infant hunger cues

Maternal perceptions of infant hunger cues were investigated by a self-administered questionnaire. A multiple choice question was used to ask “What are signs of your infant’s desire to feed?” The options provided were based on the literature and expert recommendations and included hand sucking, drooling, mouth opening, crying, and moving head frantically from side to side, other behaviors, and not clear. The mother chose one or more options according to the actual situation and could provide a specific text description of what is not covered in the options in “Other”. According to the previous literature [15, 24, 25], mouth opening and drooling act as early hunger cues, hand sucking is an active hunger cue, moving head frantically from side to side and crying are late hunger cues.

### Statistical analysis

Sociodemographic characteristics, daily nursing variables, and maternal perceptions of infant hunger cues in EBF and FF groups were described by frequencies and percentages. In univariate analysis, bivariate associations between the feeding methods and the maternal perceptions of infant hunger cues, including maternal perceptions of the number of infant hunger cues, and early, active, late hunger cues, were evaluated using the chi-square test.

Logistic regression was used to examine the association between maternal perceptions to infant hunger cues and the sociodemographic variables, daily nursing variables, feeding methods measures in multivariate analysis. This regression employed four models, in which the dependent variables included the number of perceived hunger cues (Model A), hand sucking (Model B), moving head frantically from side to side (Model C), and crying (Model D), respectively. And nine multi-categorical variables, including infant’s birth weight, maternal age, father’s age, maternal education level, father’s education level, family structure, location, feeding interval, and feeding duration, were transformed into dummy variables in logistic analysis. Odds ratios (ORs) were presented as results for both bivariate associations and logistic regression model. All of the data preparation and statistical analyses were performed using the SPSS for Windows software program (version 25.0).

## Results

### Demographic characteristics & daily nursing related to infant hunger cues perceptions

The sample included a total of 326 mother-infant dyads, including 188 dyads in EBF group and 138 dyads in FF group. About the number of hunger cues, 125 mothers (38.34%) perceived 1 hunger cue, 103 mothers (31.60%) perceived 2 hunger cues, 65 mothers (19.94%) perceived 3 hunger cues, 30 mothers (9.20%) perceived 4 hunger cues, and 3 mothers (0.92%) perceived 5 hunger cues. Three participants marked the “other” option (0.92%, including “Waving hands, reaching tongue, not sleeping”), and zero chose the “not clear” option (0.00%). However, the mothers who selected “other” also all perceived at least one hunger cues that this study involved. The demographic characteristics of the participants were shown in Table 1. The analysis indicated that there were no statistically significant differences in gender, ethnicity, mother’s age, mother’s education level, family structure, and one-child status between EBF group and FF group (all *p* > 0.05). When the demographic variables of the two groups were compared as stratified by region, only the study subjects in Qingdao had statistical differences in infant’s gender and infant’s ethnicity.

**Table 1:** Demographic Characteristics and daily nursing variables of the Participants

Variable	Exclusive Breastfeeding (EBF) N (%)	Formula Feeding (FF) N (%)	*χ* ^2^	*p*
**Demographic Characteristics**				
Infant’s Gender			0.51	0.48
Boy	96(51.1)	76(55.1)		
Girl	92(48.9)	62(44.9)		
Infant’s Ethnicity			$<$0.001	0.99
Han Nationality	150(79.8)	110(79.7)		
Minority Nationality	38(20.2)	28(20.3)		
Mother’s Age			1.82	0.40
≤ 24	15(8.0)	14(10.1)		
25–35	152(80.9)	103(74.6)		
≥36	21(11.2)	21(15.2)		
Mother’s Education Level			4.90	0.18
Completed Junior High School or Less	21(11.2)	26(18.8)		
Completed Senior High School or Technical Certificate	32(17.0)	26(18.8)		
Associate Degree	55(29.3)	39(28.3)		
Bachelor Degree or Above	80(42.6)	47(34.1)		
Family Structure			0.59	0.74
Nuclear Family	73(38.8)	50(36.2)		
Linear Family	103(54.8)	81(58.7)		
Composite/Single-parent/Reconstituted	12(6.4)	7(5.1)		
One-child Status			0.03	0.87
Yes	121(65.1)	91(65.9)		
No	65(34.9)	47(34.1)		
**Daily Nursing**				
Mother Is the Primary Caregiver			12.57	$<$0.01
Yes	182(96.8)	119(86.2)		
No	6(3.2)	19(13.8)		
Sleeping with Mother			15.15	$<$0.01
Yes	182(96.8)	117(84.8)		
No	6(3.2)	21(15.2)		
Feeding Interval (hour)			17.64	< 0.01
≤ 2	73(38.8)	24(17.4)		
3	93(49.5)	90(65.2)		
≥ 4	22(11.7)	24(17.4)		
Feeding Durations (min)			1.65	0.44
≤ 10	73(38.8)	56(40.6)		
11–20	76(40.4)	61(44.2)		
> 20	39(20.7)	21(15.2)		

Table 1 also compared the differences in daily parenting factors that may be related to perceptions of infant hunger cues in the two groups. The proportions of EBF mothers who were primary caregivers, who slept with their infants, and who fed their infants at intervals of no more than 2 h (96.8%, 96.8% & 38.8%, respectively) were higher than that in FF group (86.2%, 84.8% & 17.4%, respectively), and these differences were statistically significant (*p* < 0.05).

### Comparison of self-reported maternal perceptions of infant hunger cues between the two feeding method groups

Overall, mothers in this study chose at least one infant hunger cue, and 61.7% chose two or more infant hunger cues. The percentage of mothers who perceived two or more infant hunger cues in EBF group (66.5%) was higher than in FF group (55.1%), with statistically significant (*p* < 0.05).

**Fig. 1:**
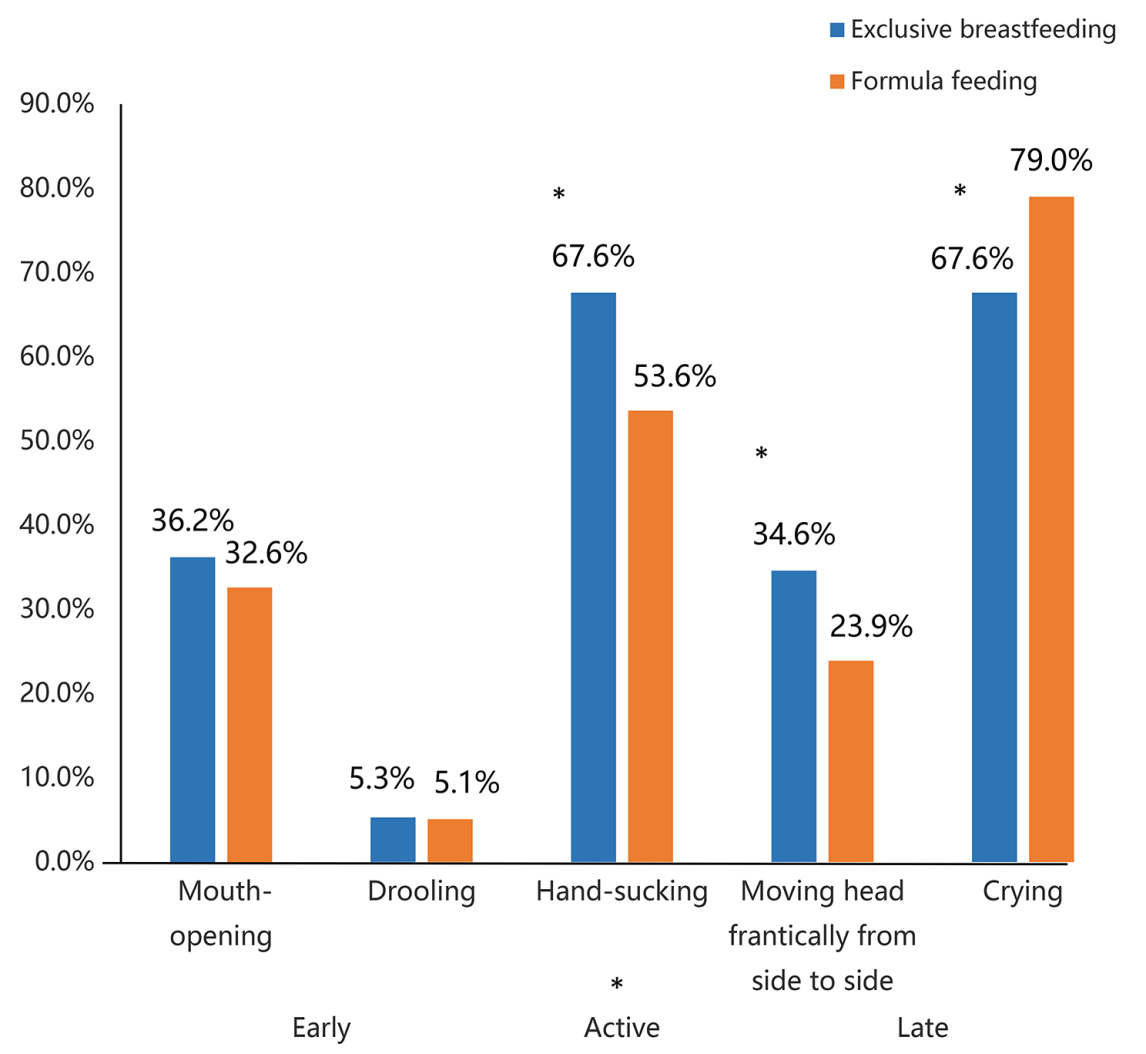
The percentage of early, active and late infant hunger cue perceived by the two groups of mothers’ self-report (*P<0.05)

Figure 1 showed the percentages of mothers who self-reported perceiving five infant hunger cues in the early, active, and late cues in EBF and FF groups. The percentages of mothers who perceived infant hand sucking and moving their head frantically from side to side as hunger cues were higher in the EBF group (67.6% & 34.6%, respectively) than that in FF group (53.6% & 23.9%%, respectively). However, the percentage of mothers in EBF group who perceived infants’ crying as a hunger cue (67.6%), was lower than that in FF group (79.0%). The above three differences were statistically significant (*p* < 0.05).

In terms of perceiving hunger cues in different periods, the percentage of mothers in EBF group who could perceive the active hunger cue (67.6%) was higher than in FF group (53.6%; *p* < 0.05). However, the differences in the percentage of EBF and FF mothers who could perceive early hunger cues (37.2% vs. 35.5%) and late hunger cues (79.3% vs. 82.6%) were not statistically significant (*p* > 0.05).

### Multivariate analysis of maternal perceptions of infant hunger cues

Before multivariate analysis, correlation analysis was performed on daily care variables. The Kaiser-Meyer-Olkin (KMO) value was 0.502 and Bartlett ‘s Sphericity Test *χ*^2^ was 94.48 (*p* < 0.01), showing that there was a correlation between variables and suitability for factor analysis. As shown in Tables 2, there was a significant correlation between “Mother Is the Primary Caregiver” and “Sleeping with Mother” (*p* < 0.01), which were selected as the interaction items for the following binary logistic regression analysis.

**Table 2:** Correlation Matrix of Daily Nursing Variables

Variable		Mother Is the Primary Caregiver	Sleeping with Mother	Feeding Interval	Feeding Duration
Mother Is the Primary Caregiver	Pearson Correlation	1	0.50	0.02	0.04
	Sig. (2-tailed)		<0.01*	0.77	0.52
Sleeping with Mother	Pearson Correlation	0.50	1	-0.01	0.07
	Sig. (2-tailed)	<0.01*		0.81	0.20
Feeding Interval	Pearson Correlation	0.02	-0.01	1	-0.02
	Sig. (2-tailed)	0.77	0.81		0.74
Feeding Duration	Pearson Correlation	0.04	0.07	-0.02	1
	Sig. (2-tailed)	0.52	0.20	0.74	

*. Correlation is significant at the 0.01 level (2-tailed)

Binary logistic regression analysis (independent variable entry level = 0.05, elimination level = 0.10) was conducted, with the sociodemographic, feeding method, and daily nursing variables as the independent variables and infant hunger cues as the dependent variables. Only hand sucking, moving head, and crying were considered in this analysis, as in the univariate analysis there was no difference between the EBF group and FF groups in mouth opening and drooling). This regression employed four models, in which the dependent variables included the number of perceived hunger cues (Model A), hand sucking (Model B), moving head frantically from side to side (Model C), and crying (Model D), respectively.

As shown in Table 3, all four models showed that mothers’ perception of infant hunger cues was related to feeding methods (*p* < 0.05).

**Table 3:** Analysis of Multi-factors Affecting Maternal perceptions of Infant Hunger Cues

the Independent Variable the Dependent Variables	Model A: based on the Number of Hungry Cues perceived by Mothers		Model B: Based on Hand-sucking		Model C: Based on Moving Head Frantically from Side to Side		Model D: Based on Crying
	*OR* (95% *CI*)		*OR* (95% *CI*)		*OR* (95% *CI*)		*OR* (95% *CI)*
Infant’s Ethnicity (base: Han Nationality)	1.63(0.63–2.15)		0.77(0.42–1.40)		1.43(0.77–2.66)		1.25(0.64–2.41)
Infant’s Gender (base: boy)	0.68(0.42–1.10)		1.11(0.69–1.80)		0.91(0.55–1.51)		0.60(0.36–1.01)
Mother’s Age (base:≤24y)							
25-35y	0.75(0.30–1.85)		0.81(0.33–1.99)		1.42(0.54–3.75)		0.55(0.19–1.59)
$>$35y	0.87(0.28–2.73)		0.55(0.18–1.72)		2.00(0.59–6.79)		0.61(0.17–2.24)
Mother’s Education Level (base: Completed Junior High School or Less)							
Completed Senior High School or Technical Certificate	1.60(0.68–3.75)		2.76(1.17–6.50) *		0.74(0.28–1.92)		1.54(0.62–3.82)
Associate Degree	3.20(1.65–7.90) *		4.20(1.85–9.55) *		0.78(0.32–1.90)		1.35(0.59–3.12)
Bachelor Degree or Above	3.01(1.34–6.76) *		2.73(1.22–6.12) *		0.88(0.37–2.15)		1.71(0.73-4.00)
Family Structure (base: Nuclear Family)							
Linear Family	1.83(1.10–3.07) *		0.83(0.50–1.40)		2.68(1.50–4.78) *		1.47(0.86–2.52)
Composite/Single-parent/Reconstituted family	0.98(0.34–2.88)		0.50(0.17–1.45)		2.82(0.93–8.57)		1.31(0.41–4.17)
One-child Status (base: No)	0.76(0.44–1.31)		0.96(0.55–1.67)		0.87(0.49–1.57)		0.85(0.47–1.51)
**Feeding Patterns (base: FF group)**							
**EBF group**	1.70(1.01–2.85) *		1.72(1.04–2.87) *		2.07(1.19–3.62) *		0.53(0.30–0.93) *
Feeding Interval(hours) (base: ≤2 h)							
3 h	1.23(0.70–2.20)		0.84(0.47–1.51)		1.79(0.95–3.36)		0.84(0.45–1.56)
≥4 h	1.54(0.70–3.44)		0.83(0.38–1.85)		1.77(0.75–4.16)		0.76(0.33–1.75)
Feeding Duration(minutes) (base: ≤10 min)							
11-20 min	1.34(0.79–2.29)		0.94(0.55–1.60)		1.37(0.79–2.39)		0.92(0.53–1.60)
≥21 min	1.23(0.62–2.43)		0.53(0.27–1.04)		1.19(0.57–2.48)		1.47(0.69–3.17)
Mother Is the Primary Caregiver*Sleeping with Mother (base: Yes)	0.96(0.65–1.40)		1.07(0.73–1.55)		0.99(0.66–1.49)		1.23(0.73–2.05)

The dependent variable were identified hunger cues (0="yes”, 1="no”) and the number of identified infant hunger cues, including overall(1="1”, 2="≥2”), Hand-sucking, Moving Head Frantically from Side to Side and Crying ,respectively (1="yes”, 0="no”). Independent variables included the gender (1="boy”, 2="girl”), infants’ ethnicity (1="Han Nationality”, 2="Minority Nationality”), mothers’ age (1="≤24”, 2="25–35”, 3="≥36”), mother’s education level (1="junior high school and below”, 2="senior high school or technical certificate”, 3="associate degree”, 4="bachelor degree or above”), family structure (1="nuclear family”, 2="linear family”, 3="composite/single-parent/reconstituted family”), one-child status(0="no”, 1="yes”), mother as the main caregiver (1="yes”, 2="no”)*infant sleeping with mother(1="yes”, 2="no”), feeding interval (1="≤2 hours”, 2="3 hours”), 3="≥4 hours”), feeding duration (1="≤10 minutes”, 2="11–20 minutes”, 3="≥21 minutes”), and feeding pattern (0="formula feeding”, 1="exclusive breastfeeding”)

*p < 0.05

a: Model goodness of fit: -2 Likelihood = 394.93, H-L test: *χ*^2^ = 4.55, *p* = 0.81

b: Model goodness of fit: -2 Likelihood = 402.03, H-L test: *χ*^2^ = 5.38, *p* = 0.72

c: Model goodness of fit: -2 Likelihood = 353.58, H-L test: *χ*^2^ = 11.28, *p* = 0.19

d: Model goodness of fit: -2 Likelihood = 371.00, H-L test: *χ*^2^ = 8.18, *p* = 0.42

The number of infant hunger cues perceived by the mother in Model A was related to the feeding method, mother’s educational level and family structure. EBF mothers were 1.70 times more likely than FF mothers to perceive multiple infant hunger cues (95% CI: 1.01–2.85). Mothers with an associate’s degree and a bachelor’s degree or above were 3.20 times more likely (95% CI: 1.65–7.90) and 3.01 times more likely (95% CI: 1.34–6.76), respectively, to perceive multiple infant hunger cues than those with a junior high school education or less. Mothers who were living in a linear family were 1.83 times more likely than those in a nuclear family to perceive multiple infant hunger cues (95% CI: 1.10–3.07).

In Model B, the feeding method and mother’s educational level were associated with the perception of “infant hand sucking”. EBF mothers were 1.72 times more likely to perceive infant hand sucking (95% CI: 1.04–2.87) than FF mothers. Mothers with a senior high school or technical certificate were 2.76(95% CI: 1.17–6.50) times more likely to perceive “hand sucking” as a hunger cue than those with a junior high school education or less. Similar results were shown in mothers with an associate’s degree (OR = 4.20,95% CI: 1.85–9.55), and a bachelor degree or above (OR = 2.73,95% CI: 1.22–6.12).

In Model C, the feeding method and family structure were associated with a mother’s perception of “infant moving his head frantically from side to side”. EBF mothers were 2.07 times more likely to perceive the cue of “infant moving his head frantically from side to side” than FF mothers (95% CI: 1.19–3.62). Compared to mothers who were living in nuclear families, mothers living in linear families were 2.68 times more likely to perceive this cue (95% CI: 1.50–4.78).

In Model D, only feeding method was associated with mothers’ perception of infant crying. EBF mothers were less likely to perceive infant crying as a hunger cue than FF mothers (OR = 0.53, 95% CI: 0.30–0.93).

## Discussion

This study mainly explored the differences in perception of infant hunger cues in Chinese mothers with 3-month-old infants under different feeding methods. The results of this study provided preliminary evidence for understanding mothers’ perceptions of infant hunger cues in the Chinese population, which will provide a support to promoting early childhood responsive feeding.

We found that the breastfeeding might support mothers to perceive infant hunger cues. This result is consisted with the previous researches [16, 36].

This study further explored the maternal perceptions of infant hunger cues under different feeding methods. As presented in the results, mothers in EBF group and mothers in FF group differed in their perceptions of the number of hunger cues, infant “sucking hands”, and “moving their heads frantically from side to side”, which supports the notion that mothers’ perception of infant hunger cues differs across feeding methods. Mothers in the EBF group perceived more infant hunger cues than mothers in the FF group, proved that breastfeeding may establish mothers’ higher levels of sensitivity to infants’ needs, especially in the first three months of life [44, 45]. Breastfeeding mothers cannot always assess the amount of breast milk consumed by their infants and, thus, must pay more attention to the hunger cues sent by the infants to determine when to feed. FF mothers, in contrast, have easier access to milk intake information and may disregard infant hunger cues, especially when hunger cues are inconsistent or unclear [46, 47]. It is also possible that breastfed infants had a higher level of engagement with hunger cues [16], and a more positive mealtime experience than formula-fed infants [36], and produced more frequent hunger cues [15]. In addition, infant-led feeding approaches (associated with breastfeeding) were related to higher awareness of infant hunger cues [48].

Interestingly, mothers in the breastfeeding group had lower perceptions of infant crying than mothers in FF group. This may be due to crying is a late hunger cue, and breastfeeding mothers were more in tune with their baby’s cues during feeding [36]. It also might be the differences in the locus of control in the feeding [49], that satiating FF is due to volume compared to the frequency of breastfeeding. Mothers who breastfed have less control over feeding behaviors [1]. Breastfed infants may be more satisfied and they may not cry as often because they are in charge of when the meal is initiated [16]. It should be noted that crying is not a specific feeding cue [25], when the infant is abnormally crying, non-starvation reasons should be considered and actively seeking medical treatment.

This study also explored maternal perceptions of infant hunger cues for different stages of feeding. There were high or low perceptions of different hunger cues, and there was still room for improvement in the mothers’ perceptions of infant early hunger cues. Hodges et al.(2016) found that, from 3 to 18 months, mouth opening was frequent and predominated at each time point [25]. However, the results of this study showed that the perceptions of infant hunger cues were mainly concentrated in the active and late stages in mothers in the EBF and FF groups, and the perceptions of early hunger cues (“mouth opening” and “drooling”) were lower in both groups. It may be because early hunger cues are less intense than in the active and late stages [27], early hunger cues were relatively rare [25], or because the mother did not realize that these cues represent hunger in real feeding situations [16]. The current lack of guidance on infant early hunger cues in health care services in China may be the most important influencing factor. In addition, consideration must be given to the fact that mother’s failure to use early cues may not reflect a mistake on her part. It could be a result of a faulty regulatory mechanism in the baby; no appropriate cues were available as feedback [24]. Or because mothers need a certain amount of time to perceive infant hunger cues, and tend to ignore the early hunger cues. Further exploration is necessary in this regard.

Regarding the multifactor analysis of maternal perceptions of infant hunger cues, we found that maternal education level and family structure were associated with the mother’s perception of infant hunger cues, a point that deserves further attention. Gross et al.(2010) similarly found that maternal education level and feeding methods were associated with infant hunger cues perceptions [23]. In our study, mothers with higher education levels and those in multigenerational families were more likely to perceive infant hunger cues, likely because more educated mothers tend to have higher cognitive and health management abilities. They also enjoy more socio-economic advantages, which can help them to obtain health knowledge and services [50–53], and better mastery of the child’s health knowledge and health awareness [54]. For these reasons, maternal education level may be an especially important factor impacting feeding methods, particularly since most primary caregivers of infants in this study were mothers (92.33%). The direct and indirect roles played by maternal education level should be further explored in future studies. In terms of the impact of family structure (i.e., with grandparents, parents, and children living together) can allow parents to receive childrearing help from grandparents, thereby reducing the stress they face [42]. Parents living in linear family structures may also be able to benefit from the parenting advice and lived experiences of grandparents. For these reasons, linear family structures can allow mothers to focus more time and energy on their children. These factors suggest that child healthcare providers need to provide more targeted and specific guidance to mothers with lower education level, living in nuclear families, and FF to improve their perceptions of infant hunger cues and to promote responsive feeding.

### Limitations

This study is the first to explore maternal perceptions of infant hunger cues under different feeding methods in a Chinese population, however, there are still some potential limitations. First, this study used self-reported data reflecting maternal perceptions of infant hunger cues, which may be affected by selective reporting or recall bias. The research group also collected videos to objectively observe the mother-infant feeding process so that they can be analyzed in subsequent studies. Additionally, mothers perceiving hunger cues does not necessarily imply that they promptly and adequately respond to these cues, which is an important additional component of responsive feeding. The potential disconnect between perception of hunger cues and correct response to these cues requires further study and attention. Second, this study only evaluated the common indicators of infant hunger cues, without incorporating infant satiety cues. But tried to use 5 indicators at different levels of early, active and late to reflect the mother’s perception, and will further enrich the content of satiety cues in future studies. Third, the population corresponding to the sample size calculation was not entirely consistent with this study population, as the perceived rate of 3-month-old infant hunger cues by mothers was not found in previous reports of Chinese population (This study used the report of Latina mothers participating in New York City WIC programs with a singleton infant aged < 5 months). This study obtained maternal perceptions of some hunger cues of Chinese population, which provided a basis for future research. And last, some results, while technically statistically significant, had a 95% CI close to 1. We will try to use the observation data to make our conclusions stronger in subsequent studies.

## Conclusion

Mothers who use different feeding methods in China also display different levels of perceptiveness toward infant hunger cues. Exclusively breastfeeding mothers of 3-month-old infants are more likely to perceive infant hunger cues than FF mothers in China, and impacts from the mother’s educational level and family structure are also observed. It is necessary to increase the health education of infant hunger and satiety cues to caregivers in Chinese population, especially among less educated mothers, mothers living in nuclear families, and FF mothers. Targeted education interventions can improve the perception of infant hunger cues by these caregivers and may contribute to the promotion of responsive feeding practices.

## Data Availability

The datasets used and/or analyzed during the current study are available from the corresponding author on reasonable request.

## Abbreviations

MCHs: Maternal and Child Health Care Institutions
EBF: exclusively breastfeeding
